# Neuroprotective effect of Mulmina™ against chemotherapy-induced cognitive decline in normal mice

**DOI:** 10.3892/br.2021.1433

**Published:** 2021-04-28

**Authors:** Manas Kinra, Niraja Ranadive, Karthik Gourishetti, Pawan Ganesh Nayak, Rajesh N. Jagdale, Syed Mushtaq Ahmed, Kaggundi V. Raghavendra, Jayesh Mudgal, Krishnadas Nandakumar

Biomed Rep 14: Article no. 1, 2021; DOI: 10.3892/br.2020.1377

Following the publication of the above article, the authors have realized that the title appeared incorrectly: “rats” appeared in the title erroneously, and the title should have read as “Neuroprotective effect of Mulmina™ against chemotherapy-induced cognitive decline in normal **mice**”. This error was not picked up upon during the process of correcting the proofs; the corrected title is now featured above.

The Editor regrets that this error was not corrected before the paper was sent to press, and apologizes to the authors for the inconvenience caused.

